# Recent Advances in the Copolymerization of Ethylene with Polar Comonomers by Nickel Catalysts

**DOI:** 10.3390/polym14183809

**Published:** 2022-09-12

**Authors:** Randi Zhang, Rong Gao, Qingqiang Gou, Jingjing Lai, Xinyang Li

**Affiliations:** Department of Polyethylene, SINOPEC (Beijing) Research Institute of Chemical Industry Co., Ltd., Beijing 100013, China

**Keywords:** late transition nickel catalyst, polar monomer, functionalized polyolefin

## Abstract

The less-expensive and earth-abundant nickel catalyst is highly promising in the copolymerization of ethylene with polar monomers and has thus attracted increasing attention in both industry and academia. Herein, we have summarized the recent advancements made in the state-of-the-art nickel catalysts with different types of ligands for ethylene copolymerization and how these modifications influence the catalyst performance, as well as new polymerization modulation strategies. With regard to α-diimine, salicylaldimine/ketoiminato, phosphino-phenolate, phosphine-sulfonate, bisphospnine monoxide, *N*-heterocyclic carbene and other unclassified chelates, the properties of each catalyst and fine modulation of key copolymerization parameters (activity, molecular weight, comonomer incorporation rate, etc.) are revealed in detail. Despite significant achievements, many opportunities and possibilities are yet to be fully addressed, and a brief outlook on the future development and long-standing challenges is provided.

## 1. Introduction

Since the seminal work of Ziegler and Natta and its contribution to olefin polymerization catalysis, the research on olefin polymerization has attracted much attention from a wide range of academic and industrial communities [1,2]. Polyolefins, with an annual production of 180 million tons [3,4,5], are widely used in people’s lives such as films, pipes, sheets, packaging, electronics, medical, and military, occupying a pivotal role in modern society. Due to its excellent combination of chemical and physical properties and its low cost and good processability, many efforts have been made to further improve their properties through the controlled incorporation of polar functionalities into the otherwise nonpolar polymer structure. The introduction of a small amount of polar and functionalized groups (even 0.5 mol%) can effectively alleviate the disadvantages of most types of polyethylene (PE) [6], e.g., poor surface property and difficulties in adhering to polar materials, resulting in improved and tunable product performance, such as adhesion, barrier and surface properties, dyeability, printability and polymer miscibility, as well as increasing their business values [7].

Functionalized polyolefins have been industrially manufactured by post-polymerization functionalization and free-radical copolymerization of olefins with polar monomers, while the former requires specially designed polar monomers and a tedious synthetic procedure [8], and the latter needs harsh conditions along with poor control over the copolymer composition and microstructure [9]. In contrast, the single-site transition metal-catalyzed coordination polymerization, as the most straightforward method, can undergo mild reaction conditions with fewer side reactions (e.g., cross-linking or degradation) and can effectively control the structure of the polymer chains (including the microstructure, stereo-regularity, topology, and incorporation rate) through various catalyst structures and polymerization conditions [10,11]. However, due to a series of issues termed the “polar monomer problem” [12], no commercial process currently exists for the coordination polymerization method. For the most extensively studied and industrially relevant early transition metal catalyzed systems, the early transition metals (titanium, zirconium, chromium, etc.) are highly acidic in nature and do not tolerate functional groups, resulting in poisoning from the Lewis basic polar groups (X) and some X-related side reactions that can deactivate the catalysts [3,12]. 

**Scheme 1:**
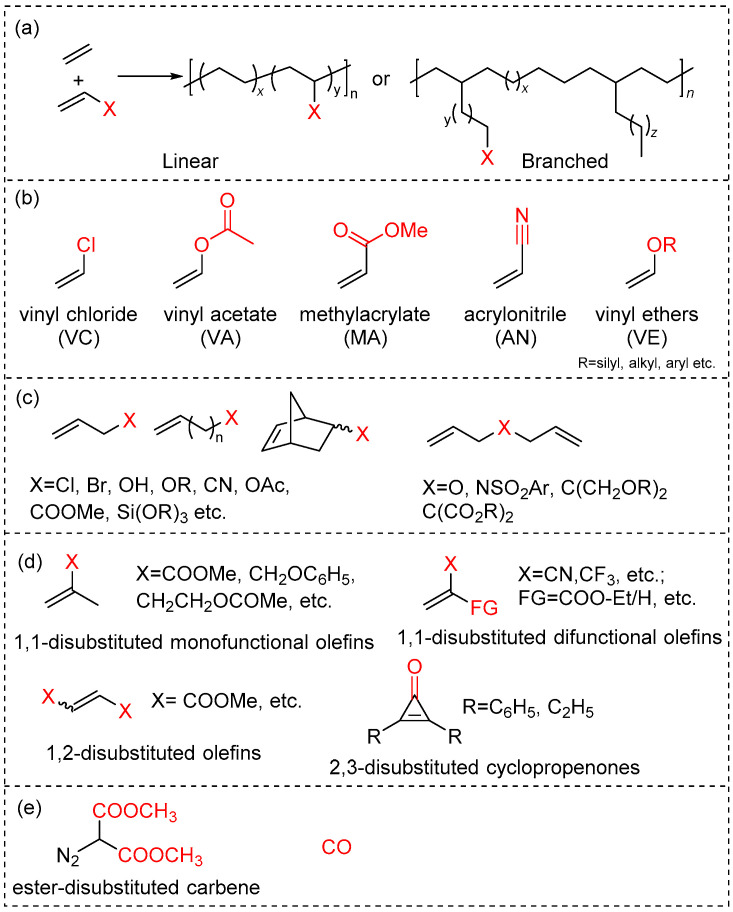
(**a**) Copolymerization of ethylene and polar monomers catalyzed by late transition metal and Common polar monomers involving (**b**) polar vinyl monomers; (**c**) polar allyl/norbornyl monomers and monomers with methylene spacers; (**d**) disubstituted monomers; (**e**) C1 monomers such as ester-disubstituted carbene and carbon monoxide (CO).

Late transition metal catalysts, especially palladium catalysts, have received attention because of their unique catalytic properties, low oxophilicity and outstanding tolerance toward polar groups that allow for direct copolymerization of ethylene and polar monomers (Scheme 1a) [3,13,14,15,16,17,18]. It is highly desirable from an industrial perspective in functional materials with commercially available and fundamental comonomers that the polar functional group directly attached to the *C=C* double bond, including acrylates, acrylonitrile, vinyl acetate, vinyl fluoride and vinyl ethers, as well as others (Scheme 1b). Special polar monomers, such as polar allyl/norbornyl monomers, monomers with methylene spacers, disubstituted monomers and C1 monomers (Scheme 1c–e), could also be employed, and the group **c** polymers are relatively easy to copolymerize with olefins due to the presence of polar groups at a remote position. 

**Scheme 2:**
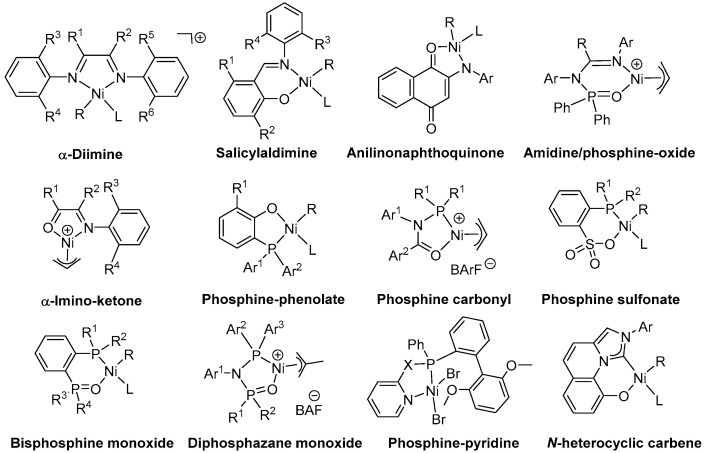
Various backbones of late transition nickel catalysts for olefin copolymerization with polar monomers.

Nevertheless, the high cost and relatively low activity of palladium catalysts limit their practical utility, and their replacement with less expensive catalysts, such as nickel catalysts, would therefore be highly competitive [19,20]. Several nickel catalysts have been explored in recent years, with ligands that include α-diimine [21], α-imino-ketone [22], salicylaldimine [23], phosphine-phenolate [24], phosphine sulfonates [25], bisphosphine monoxide [26], *N*-heterocyclic carbene [27], and other specially designed ligands [10,11] (Scheme 2). There has been increasing research interest in nickel-mediated copolymerization of olefin with polar monomers, most of which rely on the manipulation of the electronic and steric effects of ligands. Further modification to these catalysts by Brookhart, Jordan, Nozaki, Shimizu, Carrow, Chen, Cai, Jian, Li, etc., led to enhanced catalytic properties. Reviews have been extensively summarized based on catalyst classifications by Chen [11,28,29,30], Jian [10,31,32], Nozaki [2,33], Li [34], Cao [35], etc., including advanced catalytic tuning strategies containing external stimuli [36] and secondary interactions [37,38,39,40], the coordination–insertion mechanism [3,41] containing the monomer poisoning effect [42] and the functions of custom-made polar monomers [31,32]. Except for the most challenging substrates such as fundamental polar monomers, a variety of nickel catalysts with bidentate ligands have also been developed for the effective copolymerization of olefins with non-vinyl polar monomers [33], including disubstituted ethene comonomers, ester-disubstituted carbene and CO [43,44,45,46], e.g., the nonalternating copolymerization of ethylene with CO to incorporate in-chain keto groups in polyethylene chains with high molecular weight while retaining desirable material properties [44]. 

For the genuine potential in industrial applications displayed by nickel catalysts, this review will mainly discuss nickel catalysts used in the copolymerization of ethylene and polar monomers to achieve the functionalized polyolefins. Herein, we collect together the advanced results in the past three years with the main emphasis being placed on the different types of ligand structures including α-diimine, salicylaldimine, phosphine phenolates, phosphine sulfonates, bisphosphine monoxide, *N*-heterocyclic carbene and other unclassified chelates, along with the new strategies for catalyst design such as steric and electronic adjustment, secondary metal cation effects and heterogeneous strategy. The related palladium-catalyzed copolymerization would be also included if necessary for comparison. We hope our collected findings will illustrate the value of the research and furthermore will encourage other researchers to become involved in the challenges ahead. 

## 2. α-Diimine Nickel Catalysts

Since Brookhart and coworkers reported that α-diimine Ni(II) and Pd(II) complexes exhibited a unique chain-walking mechanism in olefin polymerization and copolymerized specific polar monomers which poison Ziegler–Natta/metallocene catalysts, numerous studies have been further explored focusing on ligand design including backbones and substituent modifications. One of the key characteristics of these α-diimine catalysts is enabled to undergo facile *β*-hydrogen (*β*-H) elimination/reinsertion during polymerization [28,47]. The palladium catalysts can copolymerize with various acrylates, acrylic acid and a few other polar monomers, affording various branched functionalized polyolefins with polar groups located mainly at the ends of branches [21,31,48,49,50,51,52,53,54,55,56,57,58,59,60], as well as catalyzing alternating copolymerization of substituted styrene comonomers and CO in a living fashion [61,62]. Nickel catalysts, however, are much more oxophillic and less tolerant towards polar monomers, thus always deactivated in the presence of commercial polar comonomers [2,28]. Only those comonomers bearing a spacer between the *C=C* bond and polar group could be effectively incorporated. Examples for the polymerization of ethylene with MA using classic α-diimine nickel catalysts showed that it required very harsh conditions (120 °C and 340 bar ethylene pressure) [10] that could help in the dissociation of the carbonyl coordination from the electrophilic Ni center, resulting in moderately linear (30 Me per 1000 carbons) to highly branched with predominantly in-chain acrylate incorporation (ca. 1%). In this case, the utilization of low-cost nickel α-diimine catalysts to prepare functional polymers faces fascinating challenge in this field, and many efforts have been made to design new catalysts [21,63] and new modulation strategies including multinuclear [34,48,50] catalysts and heterogeneous systems [64]. Here, we will summarize some recent major advances concerning catalyst developments that allowed far greater control over the polymerization process as well as polymer microstructures.

**Figure 1:**
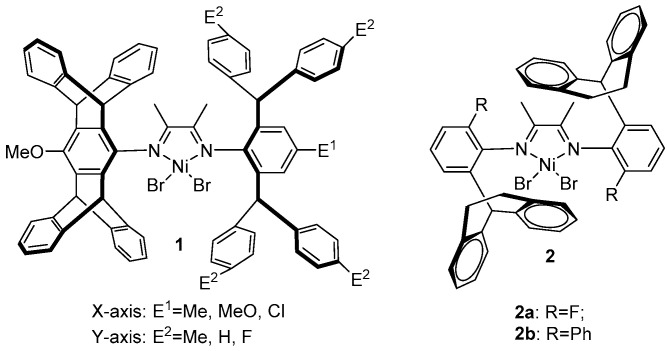
α-Diimine Ni(II) catalysts **1** and **2a**,**b** from Jian group.

Significant advances have been made in the design and modification of the ligand structures including modifications of the *N*-aryl substituents, the ligand backbone [65], desymmetrization, and the utilization of multinucleated ligands [28]. The Jian group designed a family of sterically bulky pentiptycenyl/dibenzhydryl substituted unsymmetrical α-diimine Ni(II) catalysts **1** (Figure 1) and systematically disclosed the electronic effects from both the *para*-position of *N*-aryl group (horizontal axis: Me, MeO, and Cl) and the *para*-position of dibenzhydryl moiety (vertical axis: Me, H, and F) [66,67]. In the ethylene copolymerization with methyl 10-undecenoate (UCOOMe), the electron-donating Me group concurrently on horizontal axis and vertical axis behaved the best concerning catalytic activity (38.9 × 10^4^ g mol^−1^ h^−1^), molecular weight (374 kDa), and comonomer incorporation (0.11 mol%) together; however, the electron-withdrawing group was even inactive for ethylene copolymerization. Jian et al. also constructed “sandwich-like” α-diimine nickel catalysts **2a**,**b** (Figure 1) by installing the rotationally restrained benzosuberyl substituents into the *ortho*-positions of *N*-aryl rings [68], as another way to shield the axial sites, thus improving polymerization performance. By mediating the copolymerization of ethylene with UCOOMe, **2b** gave the highest copolymer molecular weight (88 kDa), along with the highest incorporation (2.0 mol%). 

**Figure 2:**
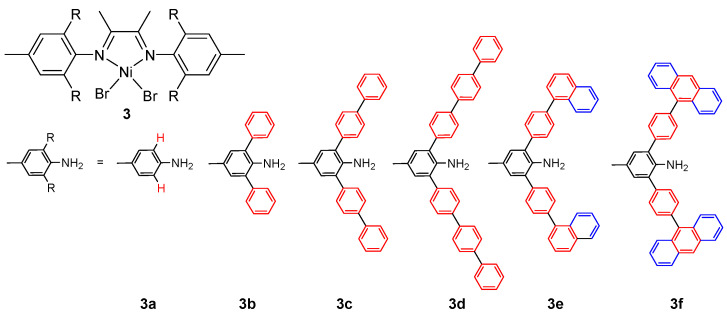
α-diimine nickel catalysts **3a**–**f** with a bi-directional steric environment on the axial sites of the active species.

Jian and coworkers further explored a novel steric strategy that imparts a bi-directional steric environment on the axial sites of the active species from both the horizontal and vertical directions (Figure 2, **3a**–**f**). In the copolymerization of ethylene with biorenewable polar monomer UCOOMe, **3f** provided the highest copolymer molecular weight and gave the lowest incorporation of co-monomer, which shed light on the steric effect on olefin copolymerization [69]. 

**Figure 3:**
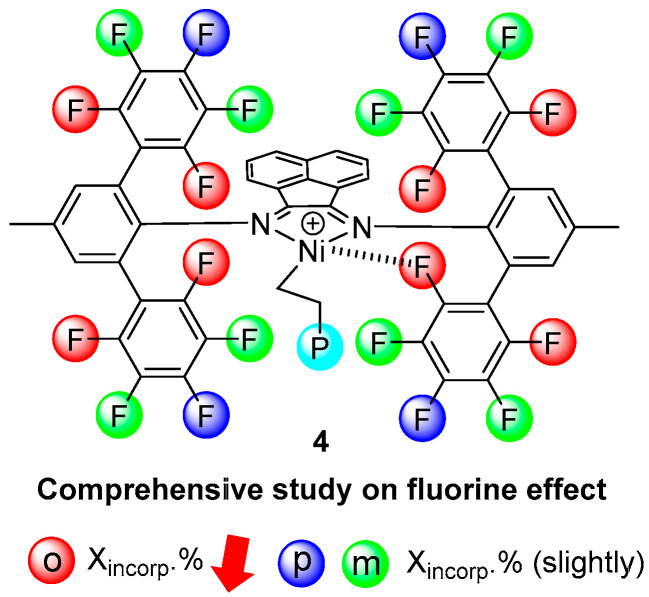
The fluorinated α-diimine Ni(II) catalysts **4** (in the copolymerization of ethylene with polar monomer, the *ortho*-F substituent is definitely unfavorable for the incorporation, and both the *meta*-F substituent and the *para*-F substituent show a slight influence in terms of incorporation).

They also reported a new series of distinct fluorinated α-diimine nickel catalysts **4** used in copolymerization with polar monomers (Figure 3), showing that the acid functionalized 10-undecenoic acid (UCOOH) behaved better than the ester functionalized UCOOMe (catalytic activity and comonomer incorporation). Although the incorporation of polar monomer was relatively low, it was enough to alter the surface property of polyethylene by the detection of water contact angles [70]. 

**Figure 4:**
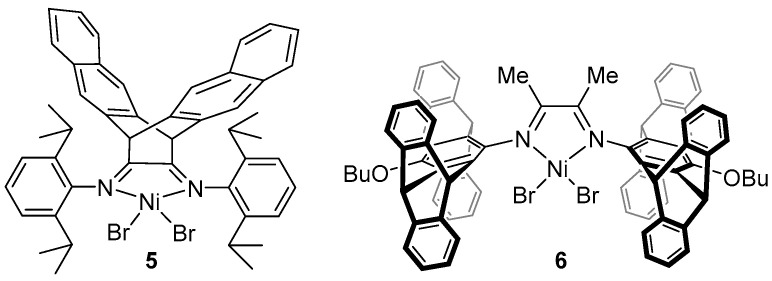
Dinaphthobarrelene backbone-based α-diimine nickel catalyst **5** and bispentiptycene-diimine nickel catalyst **6**.

The Gao group synthesized a novel dinaphthobarrelene-based α-diimine Ni catalyst **5** (Figure 4) from the viewpoint of three-dimensional (3D) space for ethylene (co)polymerizations [71], in which the 3D-constrained space around Ni center could lead to enhancement on thermal stability, living fashion, and tolerance towards polar groups in copolymerizations of ethylene with bromo-1-olefins and hydroxy-1-olefin. Even at 80 °C, copolymerization of ethylene and 10-undecenol (UOH) was still active to produced copolymer with high incorporation of 5.21 mol%, and the living copolymerization could also be realized below 35 °C, which was one of the rare samples for living copolymerization of ethylene and polar monomers.

In order to obtain more shielded Ni centers with potentially higher catalytic activities, Plenio et al. synthesized a series of bowl-shaped bispentiptycene-diimine nickel catalyst **6** (Figure 4), which could efficiently copolymerize ethylene with polar monomers UCOOMe, UCOOH, 11-chloroundecene (UCl) and 5-hexene-ol (HO), leading to polymers with up to 4.2 mol% of incorporated polar monomer [72]. The activity of the catalyst critically depended on the molar ratio of Et_2_AlCl activator and the polar functional group.

**Figure 5:**
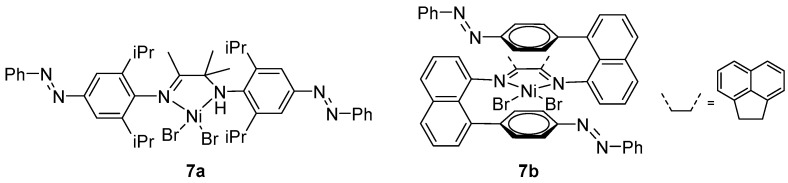
The azobenzene-functionalized nickel catalysts **7a**,**b**.

In terms of the polymerization process using external stimuli, Chen et al. designed two photoresponsive olefin polymerization systems by modulation of the ligand backbone from α-diimine to imine-amine and relocation of the azobenzene unit covalently farther from the metal center (Figure 5). The nickel catalysts **7a**,**b** were able to mediate efficient ethylene copolymerization with polar comonomers including vinyl trimethoxysilane (VTMOS), 6-chloro-1-hexene (HCl), and UOH. Attributable to a light-induced ligand electronic effect, all catalysts showed decreased activity and polymer molecular weight along with an increased polymer branching density under UV light irradiation, offering a simple and non-invasive light-controlled strategy in transition-metal-catalyzed polymerizations [73]. 

**Figure 6:**
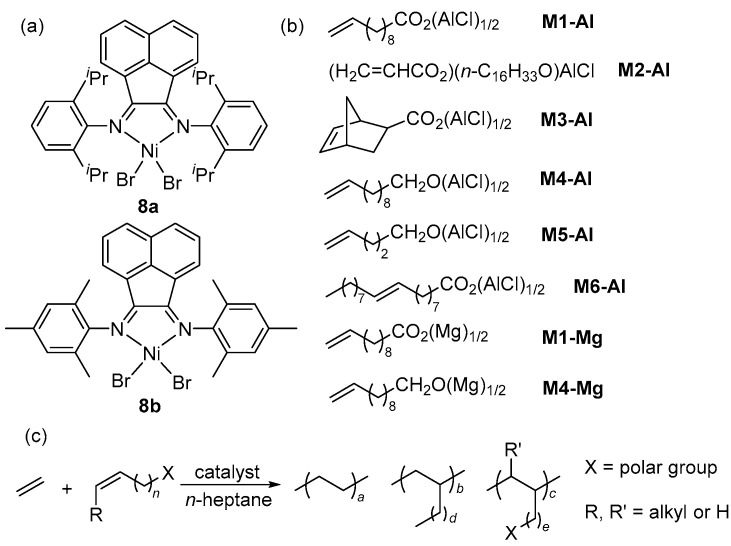
(**a**) Nickel catalysts **8a**,**b** for olefin copolymerization; (**b**) different metal–salt-based polar monomers; (**c**) ethylene copolymerization with polar monomers.

An ionic cluster strategy was designed by Chen and coworkers to control the product morphology during the synthesis of polar-functionalized polyolefins via precipitation polymerization. In classical α-diimine nickel-catalyzed ethylene copolymerization (Figure 6, **8a**,**b**), the utilization of metal–salt-based comonomer **M1-Al** displayed high catalytic activity (8.2 × 10^5^ g mol^−1^ h^−1^), generating a high-molecular-weight copolymer (*M*_w_ = 400.7 kg·mol^−1^) with a high comonomer incorporation ratio (9.6 mol%). Even at 150 °C, the copolymerization activity could reach 1.4 × 10^5^ g mol^−1^ h^−1^ with a high polar monomer incorporation ratio (57 mol%), which showed tunable mechanical properties and great potential as compatibilizing agents for mixtures of polyolefins [64]. The heterogeneous strategies could also be found in covalently immobilized multiwalled carbon nanotube (MWNTs)-supported α-diimine nickel catalysts, exhibiting high copolymerization activity and monomer incorporation ratios in the copolymerization of norbornene and *n*-butyl methacrylate [74]. 

α-diimine nickel catalysts have a considerable track record for ethylene polymerization for their excellent control of catalytic performances and improved thermal stability. In addition to their ease of synthesis and structural versatility, the chain walking capability represents one of their most fascinating features [28]. For the studies related to copolymerization, however, the existing progress is not satisfactory compared with the corresponding palladium catalysts, such as the lack of mechanism studies at molecular and electronic levels [75]. In this way, more studies need to be carried out on nickel-catalyzed copolymerization, especially to find new strategies to broaden polar comonomer substrate scopes that are not limited to the ones bearing long spacers between the double bond and acrylate group.

## 3. Salicylaldimine/Ketoiminato Nickel Catalysts

In the 2000s, neutral salicylaldimine nickel catalysts were reported by Grubbs et al., which were useful for the copolymerization of ethylene with polar-substituted norbornene and a few other special polar monomers producing branched copolymers [23]. They could tolerate functional groups such as ester and copolymerize ethylene with functional olefins, even the more challenging 1,1-disubstituted monomer methyl methacrylate (MMA) [76], so these catalysts were extensively studied by different research groups [34,77], including the installation of sterically bulky substituents to block the metal center and/or the design of new electron-donating ligands to reduce the electrophilicity of the metal center. A series of [N,O]-type nickel catalysts based on various new ligands, including *β*-ketoiminato [78], five-membered anilinonaphthoquinone [79,80,81], six-membered anilinoanthraquinone [82,83], anilinobenzoic acid methyl ester [84], 2-iminopyridine-*N*-oxide [85], imidazolin-2-imine [86,87], imine/phosphine-oxide [88,89], and cation-tunable nickel catalysts [90], have been further investigated in the copolymerization of ethylene/norbornene with polar monomers and alternating copolymerization of ethylene and CO [91]. Detailed mechanistic investigations have also been reported to indicate related insertion copolymerization [76]. 

**Figure 7:**
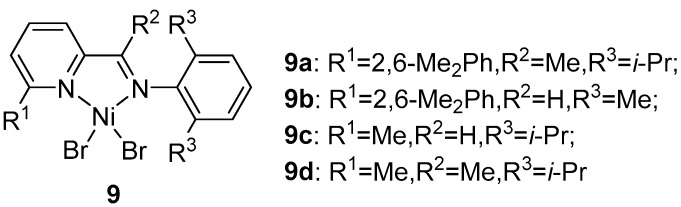
Pyridylimino Ni(II) catalysts **9a**–**d**.

Pellecchia et al. investigated a series of Ni(II) complexes **9a**–**d** (Figure 7) with four pyridylimino ligands (either aldimine or ketimine) bearing different substituents at the pyridino and at the imino moieties [92] and tested them in the copolymerization of ethylene with MA, resulting in the production of low-molecular-weight hyperbranched copolymers with MA molar contents ranging between 0.2 and 35% and inserted in a variety of modes. The method of incorporation of the polar monomer went from “in-chain only” to “everywhere but in-chain” and was dictated by both the activation mode and the solvent used to dissolve the nickel precatalyst.

**Figure 8:**
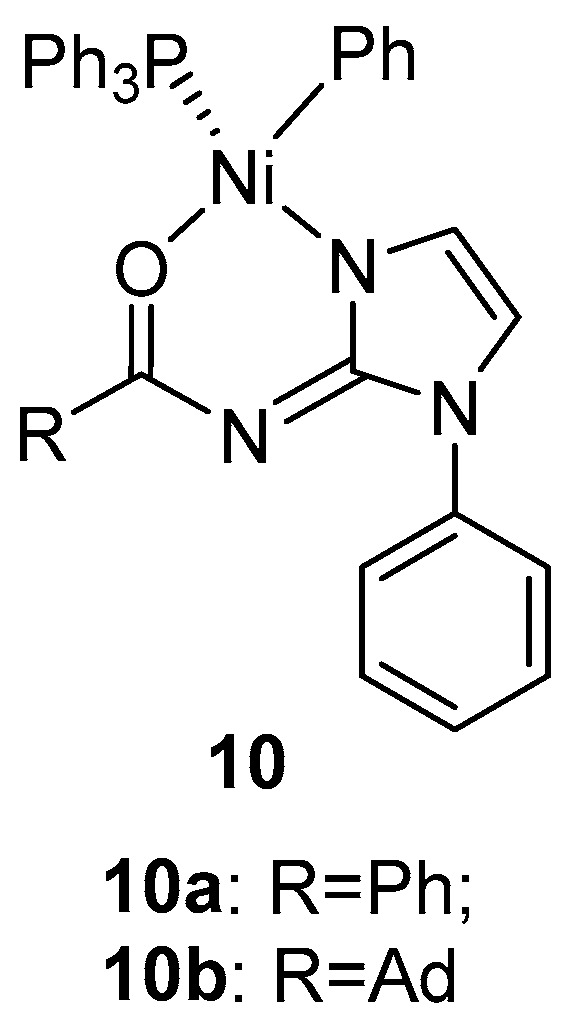
Imidazol-2-ylacylamide nickel catalysts **10a**,**b** (Ad = adamantyl).

The Cai group synthesized a novel neutral nickel complex **10a**,**b** bearing strong electron-donating *N*-acylated imidazolin-2-imine ligand (Figure 8) [93], which could conduct ethylene copolymerization with 5-hexene-1-ylacetate (HAc) with moderate activity (~10^5^ g mol^−1^ h^−1^), affording polar functionalized semicrystalline polyethylene possessing in-chain polar monomer units. The installation of the strong electron-donating framework was necessary for the effective reduction in the electrophilicity of the nickel center to enhance the tolerance of nickel complexes.

**Figure 9:**
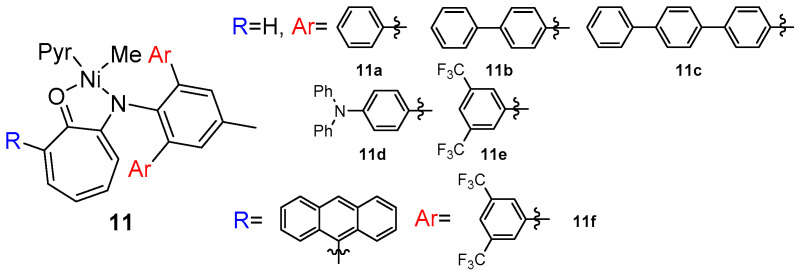
Neutral, single-component anilinotropone nickel catalysts **11a**–**f**.

Anilinotropone ligand, as the isomer of the notable salicylaldimine type, was also used in the synthesis of neutral, single-component Ni(II) catalysts **11a**–**f** (Figure 9). Compared with the classic salicylaldiminato nickel catalyst, this isomer catalyst **11f** gave 4.5-times higher TOF (5.61 × 10^3^ h^−1^), 34-times higher MW (*M*_w_ = 23.7 × 10^4^ g mol^−1^), and 1.6-times higher comonomer incorporation (*X*_M_ = 0.25 mol%) in the copolymerization of ethylene and HAc, thus enabling the formation of high-MW functionalized polyethylene [94]. Mechanistic insights further revealed both the suppression of chain transfer and promotion of chain propagation.

**Figure 10:**
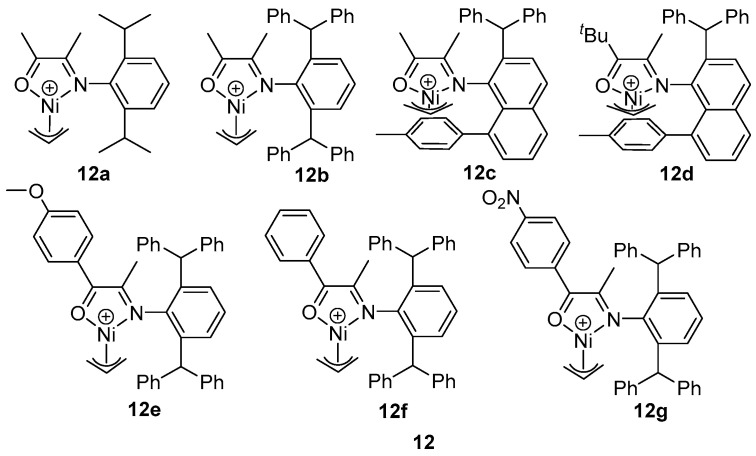
Single-component cationic α-ketiminato nickel catalysts **12a**–**g**.

The Chen group reported the copolymerization studies of a simple but extremely versatile cationic α-imino-ketone nickel system **12a**–**g** (Figure 10) with many superior properties, such as ease of synthesis, single-component nature, and simplicity in heterogenization and modification. This system demonstrated high activities (up to 2.5 × 10^5^ g mol^−1^ h^−1^), very high copolymer molecular weights (*M*_n_ up to 306,700), high comonomer incorporation (1.8–6.3%) and a high comonomer utilization ratio towards comonomers HCl and UCOOMe, owing to the sterically open nature of the α-imino-ketone system [22]. 

**Figure 11:**
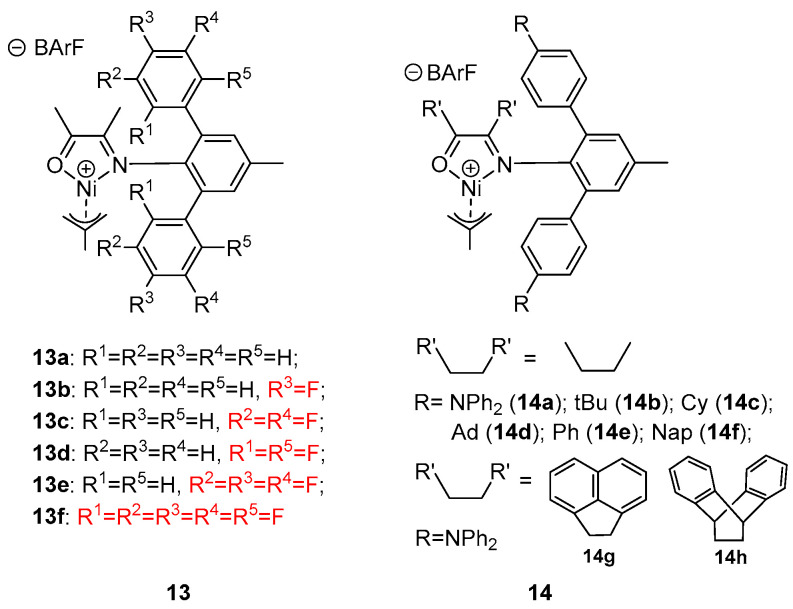
Cationic α-imino-ketone nickel catalysts **13a**–**f** and **14a**–**h** (Cy = cyclohexyl, Ad = adamantyl, Nap = Naphthyl).

Jian et al. further introduced fluorine atoms with different sites and numbers into above cationic α-ketiminato nickel catalysts to investigate the fluorine effect on ethylene (co)polymerization properties (Figure 11, **13a**–**f**). Notably, the *ortho*-fluorinated nickel catalyst **13c** showed activity and enabled the incorporation of comonomer (0.9 mol%) in E/MA copolymerization, at the same time exhibiting better copolymerization behaviors with long-chain polar monomers [95]. They also introduced different backbones and flexible and rigid axial substituents into the α-imino-ketone framework **14a**–**h** (Figure 11), and the preferred nickel catalyst **14a** exhibited high activity of 175 kg mol^−1^ h^−1^ to produce functionalized polyethylene with a molecular weight of 13.4 kg mol^−1^ and comonomer incorporation of 2.9 mol% in E-UCOOMe copolymerization [96]. 

**Figure 12:**
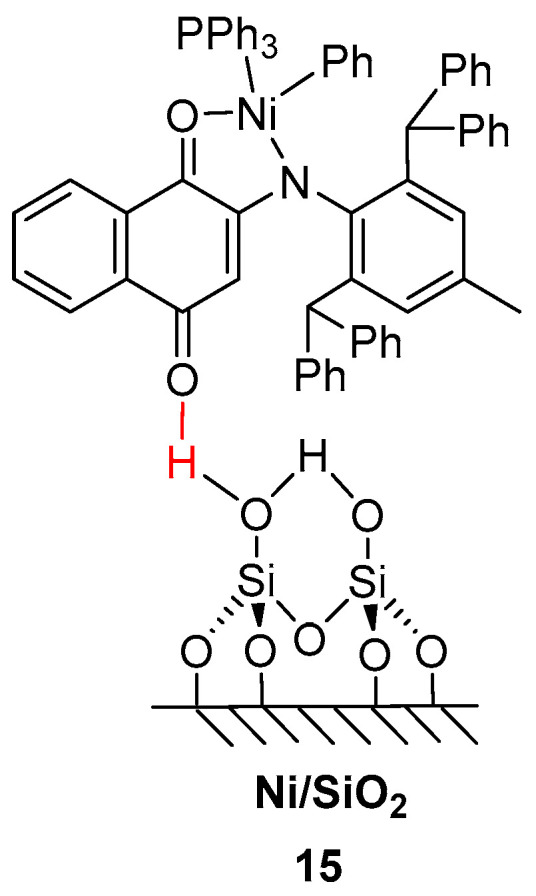
Heterogenization of anilinonaphthoquinone nickel catalysts **15**.

Due to the preference of heterogeneous catalysts in industrial olefin polymerization processes, Chen et al. recently developed a heterogeneous naphthoquinone-based [79] nickel (**Ni**/SiO_2_) catalyst **15** through hydrogen bonding interactions of the ligands with the silica surface (Figure 12) [97]. **Ni**/SiO_2_ exhibited high activities (up to 2.65 × 10^6^ g mol^−1^ h^−1^) during the copolymerization of ethylene with HAc, affording high-molecular-weight (*M*_n_ up to 630,000) polar-functionalized semicrystalline polyethylene (comonomer incorporation up to 2.8 mol%) along with great morphological control. Notably, the system showed much better properties than its homogeneous counterpart under the same conditions in both E/HAc and E/AAc (allyl acetate) copolymerization reactions.

**Figure 13:**
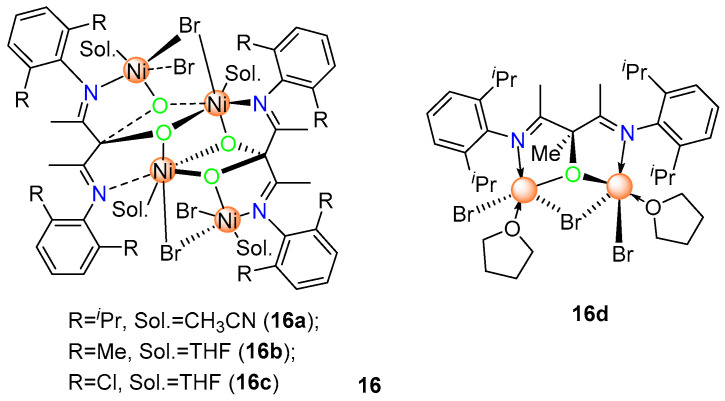
Tetranuclear nickel complexes **16a**–**c** and binuclear nickel catalyst **16d**.

Macrocyclic multinuclear [N, O] nickel catalysts have been used in ethylene or propylene [98]. Tang et al. reported a tetranuclear Ni catalyst system **16a**–**c** (Figure 13), which showed much higher efficiency in enchaining protic VA without compromising activity or *M*_w_ capability vs. its aprotic analog methyl vinyl acetate (MVA) [99]. The same trends were also seen for a range of protic monomers such as allyl acetic acid (AAA), ω-alkenoic acid, allyl alcohol and homoallyl alcohol. They further proposed a possible mechanistic scenario that involved a distinctive VA enchainment enabled by Ni···Ni synergistic effects. Based on the mechanistic insights, a binuclear nickel catalyst **16d** was then designed and proved much more efficient for the copolymerization of ethylene with either VA or acrylic acid (AA), achieving the highest TOFs so far for both ethylene and polar monomers simultaneously (e.g., 300 kg mol^−1^ h^−1^) with 2.6 mol% AA incorporation.

As described above, the cationic nickel species could copolymerize ethylene with fundamental polar monomers rather than their neutral counterparts, which are in principle more tolerant toward polar functionalities [22,95]. Further studies based the classic salicylaldimine/ketoiminato systems and their behaviors toward olefin (co)polymerization are still in progress, especially the introduction of new modulation strategies such as combination with secondary metal cations and heterogenized in situ. Advances in this area are proliferating, and we can expect new breakthroughs in the near future.

## 4. Phosphine–Phenolate Nickel Catalysts

Shell higher olefin process (SHOP) neutral nickel catalysts, discovered in the 1960s, have been successfully commercialized for the preparation of α-olefins [20,100], but are only capable of copolymerizing ethylene with polar monomers bearing functional groups remote from the olefinic double bonds. Phosphine-phenolate nickel catalysts, improved from the structurally similar SHOP type by ligand and substitutions modification, possess superior properties in dealing with fundamental polar monomers. Since Grubbs et al. reported a series of neutral nickel catalysts showing substantial tolerance toward polar agents [101], a number of effective catalysts have been developed in the following years [34,102]. Shimizu [24] and Li [103] separately described the efficient phosphine-phenolate nickel catalysts for the copolymerization of ethylene with acrylates, affording high molecular weight linear functionalized PEs. Further improvements have been made to expand the polar monomer substrate scope (e.g., acrylamide, vinyl sulfone, polyethylene glycol monomethyl ether acrylate macromonomer) and improve the properties through ligand modifications including introducing axial bulky groups [19,104]. Moreover, such nickel catalysts have successfully incorporated functionalized norbornenes into PE materials [105,106] and copolymerized propylene with some polar monomers [107]. Compared with phosphine-sulfonate and α-diimine nickel catalysts, this nickel system was an attractive choice for olefin polymerization for the unique features of good tolerance toward fundamental polar monomers, mild conditions, facile initiation, and the possibility of chain walking. Interestingly, the palladium complexes based on this type of ligand have only been shown to oligomerize ethylene [11].

**Figure 14:**
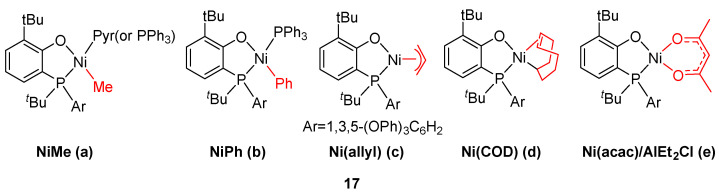
Phosphine-phenolate nickel catalysts **17a**–**e** with versatile initiating groups.

Jian et al. designed sterically bulky phosphino-phenolate Ni(II) catalysts **17a**–**e** bearing five differed “Ni-C” initiating groups including “NiMe”, “NiPh”, “Ni(allyl)”, “Ni(COD)”, and “Ni(acac)/AlEt_2_Cl”, and demonstrated the important role for initiating groups in ethylene (co)polymerization (Figure 14) [108]. Catalysts **17a** and **17b** enabled the copolymerizations of ethylene with polar comonomers such as VTMOS, HCl, and *n-*butyl allyl ether (BuAE) to give polar functionalized PEs with incorporations of up to 1.28 mol% and high molecular weights (up to 66 kDa).

**Figure 15:**
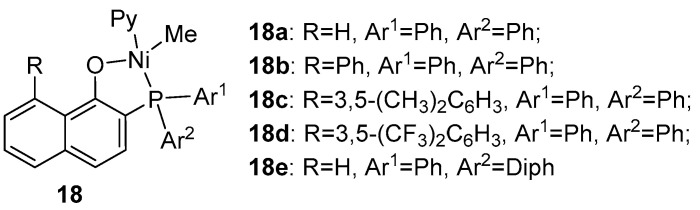
Super-robust methyl nickel catalysts **18a**–**e** with phosphino-naphtholate chelate ligands.

The Li group recently designed a series of methyl nickel complexes **18a**–**e** bearing optimized phosphino-naphtholate chelate ligands, performing unprecedented activities (up to 3.0 × 10^7^ g mol Ni^−1^ h^−1^) for ethylene polymerization (Figure 15) [109]. These super-robust nickel catalysts exhibited excellent tolerance and high incorporating capability to unsaturated acid ester without any activator under mild conditions. The catalytic activity for E/MA copolymerization was up to 3.7 × 10^5^ g mol Ni^−1^ h^−1^, and the copolymerization of ethylene with the very challenging 1,1-disubstituted difunctional dimethyl itaconate (DMI) was successfully achieved (incorporation up to 1.8 mol%; *M*_w_ up to 10,700) to give end-difunctional polyethylene with double ester groups. Based on this catalytic system, they further reported a coherent study of insertion rate, insertion mode and copolymerization reaction on various fundamental polar monomers, affording directions to rationally design catalysts. The acrylic derivatives displayed higher reactivity than non-acrylic polar monomers in the order of MA > *n*-butyl acrylate (BA) > *N,N*-dimethylacrylamide (DMAA) > MMA > vinyl triethoxysilane (VTEOS) > *N*-vinyl-pyrrolidinone (NVP), and the electron-deficient acrylic derivatives prefer 2,1-fashion while the electron-rich vinyl polar monomers favor 1,2-insertion [110]. 

**Figure 16:**
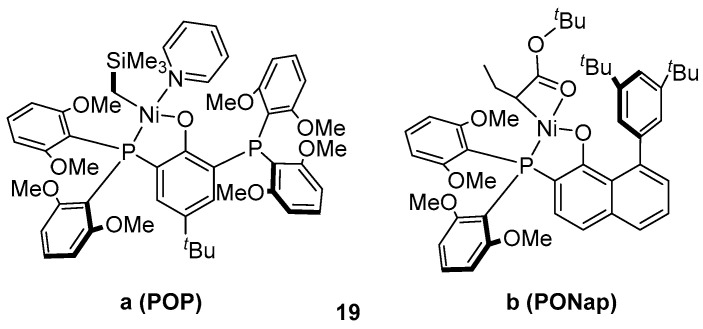
Neutral P, O-Chelated Nickel Catalysts POP-Ni-py (**19a**) and PONap-Ni-py (**19b**).

Instead of tuning steric bulk from the “P”-side, Theodor Agapie et al. reported two neutral Ni(II) catalysts **19** (Figure 16) that displayed steric bulk on the “O”-side of the phosphino-phenoxide ligand as a rotationally flexible phosphine substituent (**POP** ligand, **19a**) and a rigid aryl derivative (**PONap** ligand, **19b**) [111]. Both catalysts showed high thermal stability (highly active under temperatures up to 100 °C), enhanced *tert*-butylacrylate (*t*BA) incorporation (for **19a**: incorporation up to 12 mol%; *M*_w_ up to 94,200) and better resistance to *t*BA-induced chain transfer (for **19b**). Moreover, the nickel alkyl complexes generated after *t*BA insertion were isolated and characterized by crystallography for the first time. Synthetic, mechanistic, and DFT studies provided insights into the structural features that affect the behavior of this catalyst. 

**Figure 17:**
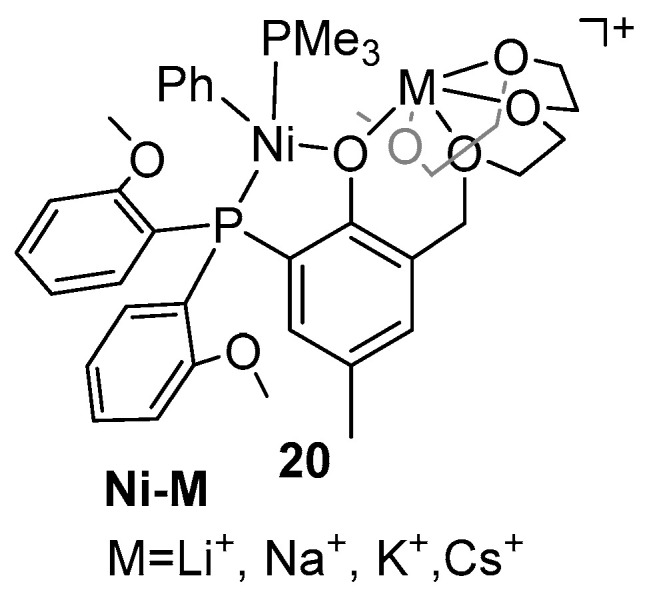
Cation-tunable heterobimetallic nickel-alkali catalyst **20** bearing phenoxyphosphine-PEG.

Do et al. developed a cation-tunable nickel phenoxyphosphine-polyethylene glycol (PEG) catalyst **20** capable of furnishing PE with distinct morphologies and demonstrated that cation tuning could readily achieve three-dimensional structures and electronic environments that are not easily accessible through conventional ligand tuning (Figure 17) [38]. While no copolymerization data were reported in this work, this tuning strategy would no doubt be highly versatile and potentially offer improved efficiency and control over existing methods [112]. 

**Figure 18:**
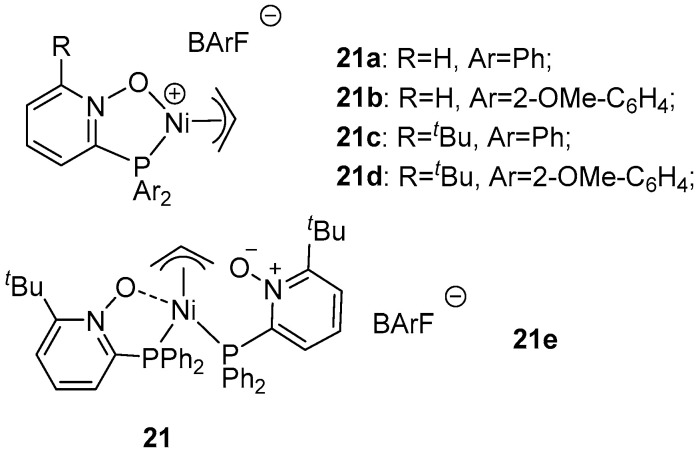
Cationic 2-phosphine-pyridine-*N*-oxide nickel catalysts **21a**–**e**.

A new type of [P, O] ligand with the combination of pyridine-*N*-oxide with phosphine as the backbone (P-O^−^) was constructed as a cationic counterpart to the phosphine-phenolate neutral system (Figure 18, **21a**–**e**) [113]. The 2-phosphine-pyridine-*N*-oxide nickel catalysts **21d** could mediate the copolymerization of ethylene with comonomer UCOOMe in high activity (up to 10^5^ g mol^−1^ h^−1^), while the corresponding palladium catalysts exhibited high activity for ethylene polymerization to produce C_4_–C_8_ products. 

**Figure 19:**
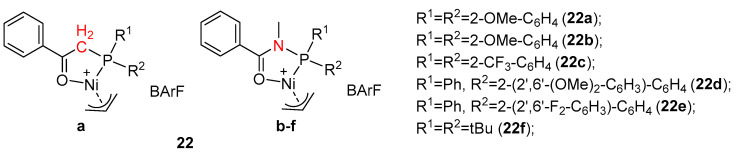
Cationic *N*-bridged phosphine–carbonyl Ni(II) catalysts **22a**–**f** bearing various electronic nature and steric bulk.

A novel family of cationic hemilabile *N*-bridged phosphine–carbonyl Pd(II) and Ni(II) catalysts **22a**–**f** bearing various electronic nature and steric bulk were then prepared by Jian and studied for ethylene (co)polymerization (Figure 19) [114]. Not only preferred cationic Pd(II) catalysts but also the more challenging Ni(II) catalysts enabled ethylene copolymerizations with challenging polar vinyl monomers such as MA, AA, and *n*-butyl vinyl ether (BuVE) with good catalytic activities, molecular weights (14.1 kg mol^−1^), and comonomer incorporation (3.3 mol%), which adding a new and effective member to the electronically asymmetric P^O-type late transition metal catalyst family. 

**Figure 20:**
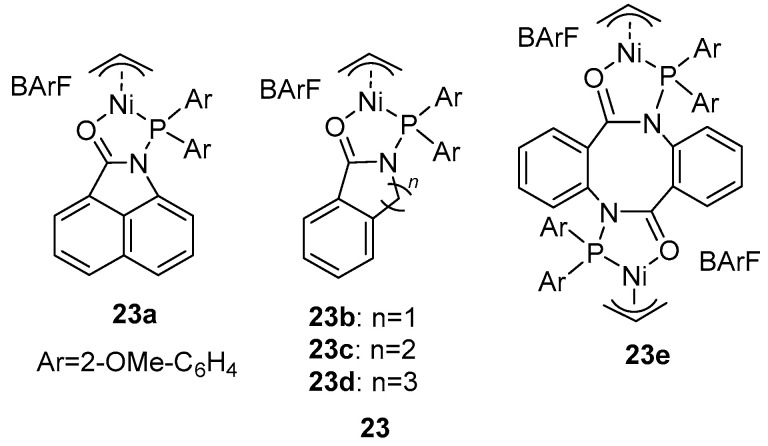
The *N*-bridged phosphine carbonyl Ni catalysts **23a**–**e** bearing five- to eight-membered-ring.

The Jian group further enhanced the system by linking the nitrogen atom with the adjacent aryl group using a cyclizing strategy and designed a series of new *N*-bridged phosphine carbonyl Ni catalysts **23a**–**e** bearing five- to eight-membered-ring structures (Figure 20) [115]. The Ni catalysts showed high performance for copolymerization with fundamental polar monomers acrylates and AA, especially for the seven-membered-ring bridged **23d**, which could be tentatively ascribed to the improved coordination strength between metal center with the hemilabile carbonyl group and the higher steric hindrance and the electron-donating nature offered by additional methylenes. 

**Figure 21:**
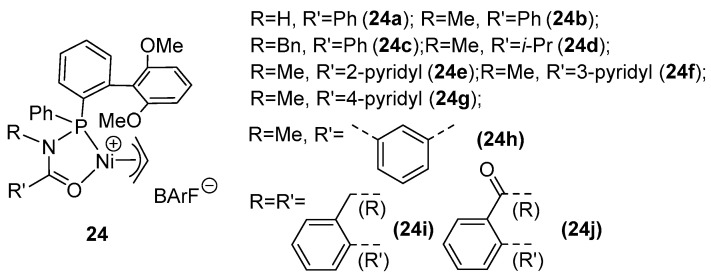
Cationic phosphine carbonyl nickel catalysts **24a**–**j** bearing various substituents on the 4ligand backbone.

Ten et al. also prepared a series of phosphine carbonyl Ni catalysts **24a**–**j** and the corresponding Pd catalysts bearing various substituents on the ligand backbone, finding that the substituent steric and electronic effects can significantly affect the properties of these catalysts (Figure 21) [116]. Both Pd and Ni catalysts could catalyze ethylene copolymerization with MA, AAc, and allyl chloride (ACl), achieving high activities as well as high polymer molecular weights (E/MA: activity up 40 kg mol^−1^ h^−1^ with 37.5 kg mol^−1^ for **24i**). 

Taken together, the bulky phosphine-phenolate nickel complex was a promising and powerful candidate for copolymerizing olefin with commercial polar monomers, except that the range of monomers applicable to this system was narrow, limiting to electron-deficient comonomers such as acrylates, acrylamides, and vinyl sulfones/sulfoxides. Compared with α-diimine Ni catalyst and the salicylaldimine/ketoiminato Ni catalysts, the phosphine-phenolate type had higher thermal stability, so copolymerization could be carried out at a higher temperature (60–80 °C). By structure adjustment from the “P” or “O”-side, a series of state-of-the-art Ni catalysts have been synthesized and extensive work is in progress on exploring the effects of various bulky substituents on catalytic activity and copolymer molecular weight as well as on broadening the scope to other commercially available polar comonomers. 

## 5. Phosphine Sulfonate Nickel Catalysts

Phosphine-sulfonate-based palladium catalysts, first reported by Drent et al. in 2002, possess unique properties that benefit the polymerization of ethylene and its copolymerization with a broad scope of polar comonomers, even including difficult candidates such as AN, VA, VE, AA and allylbenzene [117], as well as for the alternating copolymerization of vinyl monomers with CO, non-alternating copolymerization of ethylene with CO [10,14,15,17,32], and copolymerization of ethylene, carbene, and vinyl monomers [118]. The ligands combined the strong σ-donating phosphine and weak σ-donating sulfonate groups, and such an electron-unsymmetric feature was generally believed to be able to inhibit *β*-H (X) elimination, enabling consistently linear polymers that were attractive for efforts to effect stereoselective insertions of prochiral monomers [119]. Compared to catalysts ligated by α-diimine, those ligated by electronic asymmetrical phosphine-sulfonates exhibited many advantages, such as better polar monomer tolerance and thermal stability, and higher reaction temperatures (80–100 °C). In contrast to the superior properties of their palladium counterparts, phosphine-sulfonate-based nickel catalysts [25] generally led to low-molecular-weight copolymers and have not yet been reported to be active for the copolymerization of fundamental polar monomers. The structure optimization strategies around nickel catalysts were mainly based on tuning the electronic or steric effects around metal center through changing substituents at P-atom or the backbone [15,120,121,122,123,124], such as the introduction of biaryl [120] or menthyl [123] substituents, and various substituents at different ligand positions [122]. These nickel catalysts generally demonstrated high activities (above 10^6^ g mol^−1^ h^−1^) and thermal stabilities (up to 100 °C) to afford high-molecular-weight PEs (*M*_n_ up to 10^5^) toward ethylene polymerization, meanwhile exhibiting medium-to-high activities and affording improved molecular weight copolymers through copolymerization with special monomers.

**Figure 22:**
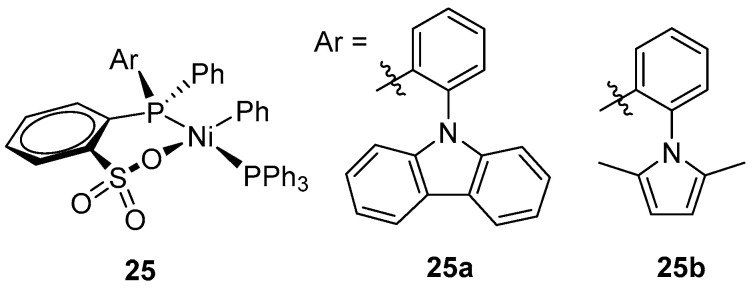
*sp*^2^ Hybrid N-based aromatic carbazolyl and pyrrolyl units substituted phosphine-sulfonate nickel catalysts **25a**,**b**.

Mechanistic insights revealed that the sterically bulky group at the axial position around nickel center is believed to suppress associative chain transfer or chain transfer to monomer [125,126]. Chen et al. recently introduced substituents bearing *sp*^2^ hybrid N atom to biaryl-derived phosphine-sulfonate nickel catalysts **25a**,**b** (Figure 22), in which the *N*-heterocyclic unit can interact with the Ni center, thus inducing ligand-metal secondary interaction. The Ni catalyst resulted in moderate catalytic activities (0.42–3.1 × 10^5^ g mol^−1^ h^−1^), low copolymer molecular weights (*M*_n_ = 2.0–2.6 kg mol^−1^) and moderate comonomer incorporation ratios (0.6–4.9 mol%) in ethylene copolymerization with comonomers HCl and UCOOMe [127]. The Chen group also enhanced copolymer performance from a comonomer perspective using 5,6-disubstituted norbornenes in palladium catalyzed copolymerization, as a highly specialty strategy that could potentially be applied to other types of transition-metal-based polymerization catalysts [128]. 

**Figure 23:**
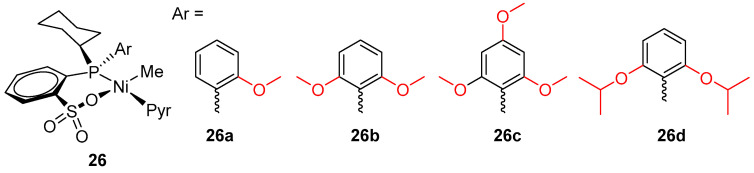
Flexible cyclohexyl/rigid aromatic phosphine-sulfonate nickel catalysts **26a**–**d**.

Jian et al. developed a series of asymmetric cyclohexyl (flexible)/aryl (rigid) substituted phosphine-sulfonate nickel complexes **26a**–**d** (Figure 23) and comprehensively identified by varying number, position, and size of alkoxy groups on aromatic substituents of phosphine. The nickel promoted ethylene copolymerization with VTMOS and HCl and gave the incorporation of 3.7 mol% and 5.0 mol%, respectively, with the highest activity of 152.0 kg mol^−1^ h^−1^ achieved by **26c** [129].

Cai et al. reported the catalytic synthesis of ammonium-functionalized polyolefins with intrinsic antimicrobial properties through palladium-catalyzed ethylene copolymerization with *N*,*N*′-dialkylimidazolium salt (ImS) functionalized α-olefins, providing ideas for the design and discovery of more functional polyolefin materials [130]. In addition, the phosphine-sulfonate palladium catalyst could achieve the direct copolymeriztion of ethylene with polar-functionalized 1,1(1,2)-disubstituted ethylenes [131,132,133] or biorenewable monomers [134], as an effective strategy to incorporate bioderived monomers into polyolefins. Though the nickel catalysts of Drent type face challenges of severer suppression of activity and relatively lower copolymer molecular weight in the presence of polar functional monomers, this less-expensive and earth-abundant catalyst is still worth exploring and the progress never ends.

## 6. Bisphosphine Monoxide Nickel Catalysts

Inspired by the superb performance of phosphine-sulfonate in the insertion copolymerization of functional olefins, Nozaki and coworkers designed cationic bisphosphine monoxide (BPMO) ligands as an alternative to mediate the copolymerization of ethylene and polar monomers [135]. The phosphine serves as a strong σ-donor, while the phosphine-oxide behaves as a weak σ-donor in BPMO ligand system. For the unique characteristics of BPMO-based Pd catalysts, such as a broad scope of commercially available polar comonomers (e.g., acrylates, acrylic acid, acrylonitrile, vinyltrialkoxysilane, allyl acetate, and long-chain comonomers), and leading to copolymers that are highly linear and have a random distribution of the polar monomer throughout the polymer chain, tremendous works have been carried out to subsequent ligand tailoring for achieving better copolymerization performance [136,137,138,139,140,141]. The optimization of catalysts mostly relied on altering steric and electronic effects of P, O donor substituents [136,137,138,139], the modifications of the ligand backbone [141,142,143], and the change in the connectivity (different linking positions) of P, O donors [140]. Interestingly, the nickel complexes developed from this type of ligands could mediate efficient copolymerization of ethylene with fundamental polar monomers such as acrylates, exhibiting comparable catalytic properties to their palladium analogues [11]. 

**Figure 24:**
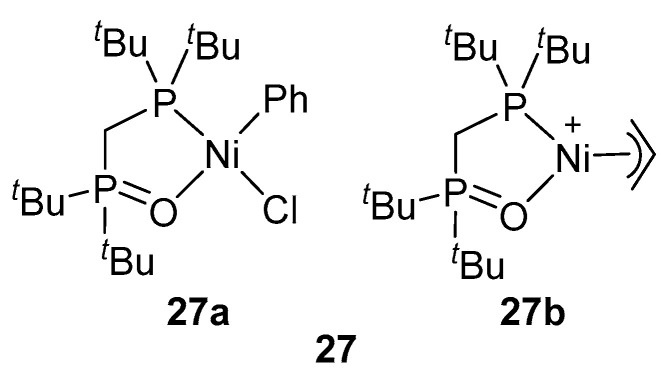
Methylene-bridged bisphosphine monoxide nickel catalysts **27a**,**b**.

Nozaki et al. applied a methylene-bridged BPMO ligand to the nickel catalyst **27a**,**b** for the copolymerization of ethylene with AAc (Figure 24). In the presence of MMAO (isobutyl-modified methylaluminoxane), high copolymerization activity (2.4 × 10^3^ g mol^−1^ h^−1^) and high acetoxy group content (0.51 mol%) could be achieved, albeit with a lower molecular weight. Notably, the in situ generated phenyl nickel chloride complex **27b** could copolymerize propylene and AAc [26]. 

**Figure 25:**
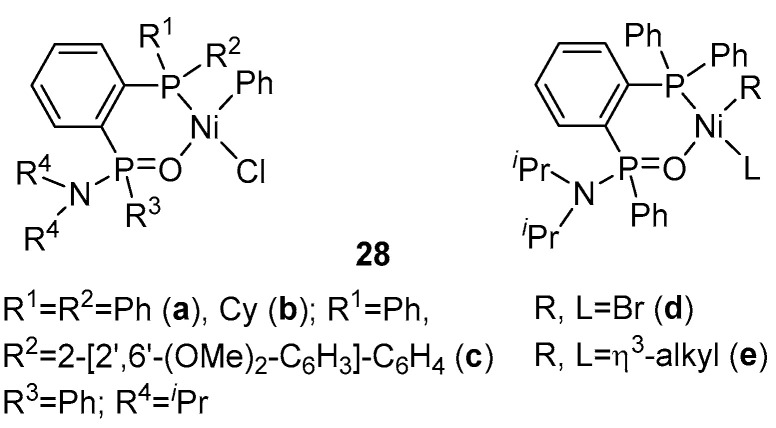
Nickel catalysts **28a**–**e** based on phosphine phosphonic amide ligands.

Chen et al. investigated the Ni catalyst **28a**–**e** (Figure 25) for the copolymerization of ethylene with polar monomers bearing a long alkyl spacer (UCOOMe, HCl and 5-acetoxy-1-pentene (PAc)), in which the four positions (R^1^, R^2^, R^3^ and R^4^) could be independently adjusted, making it highly versatile to tune the properties of the metal catalysts. The copolymerization with activities of up to 1.5 × 10^5^ g mol^−1^ h^−1^ could be achieved, along with moderate comonomer incorporations (0.2–3.3%) and moderate copolymer molecular weights (1200–27,000) [144]. 

**Figure 26:**
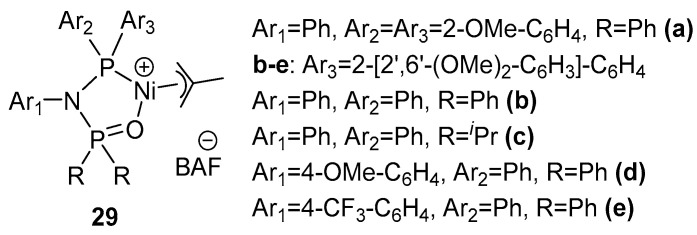
The diphosphazane monoxide (PNPO) nickel catalysts **29a**–**e**.

The Chen group recently reported a highly versatile platform based on diphosphazane monoxide (PNPO) ligands for the copolymerization of ethylene with various acrylates and vinyl ethers, in which the nickel catalysts **29a**–**e** (Figure 26) exhibited comparable behavior in these copolymerization reactions to the palladium analogues, in terms of activity, ester content, *M*_n_, and *T*_m_ in E-MA. The unique properties of this system are believed to have their origins in the shortbite-ligand platform, which increases ligand rigidity [145]. A theoretical comparison of the Drent type and PNPO catalytic systems indicated that the rigid five-membered backbone and cationic nature of the latter were beneficial for its copolymerization activity, meanwhile telling the origin of different catalytic copolymerization behaviors [146]. 

**Figure 27:**
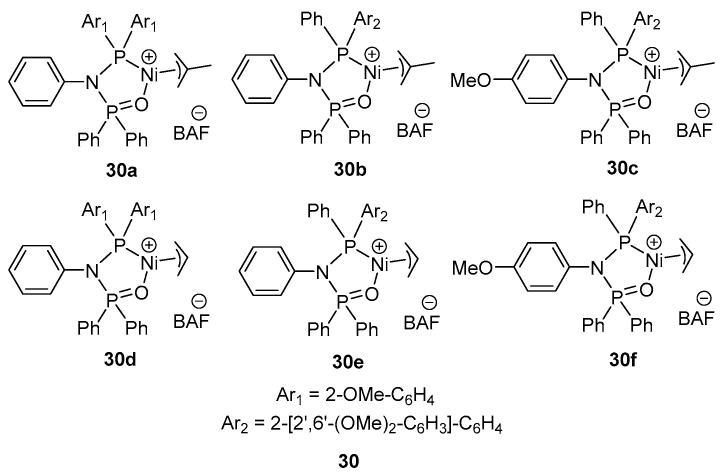
2-Methylallyl- and allyl-based nickel catalysts bearing PNPO ligands **30a**–**f**.

**Figure 28:**
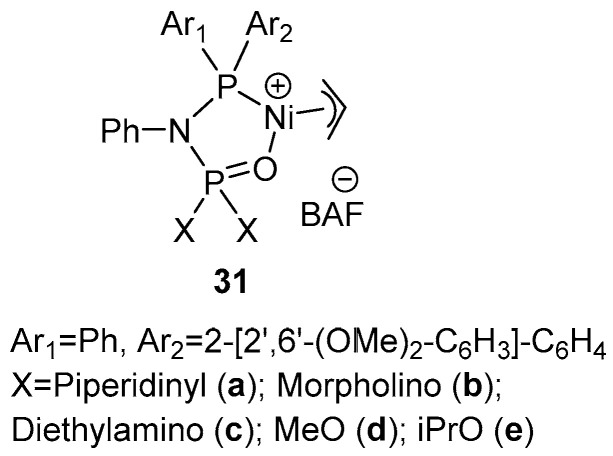
Phosphine phosphonic amide nickel catalysts **31a**–**e**.

Based on the PNPO-type ligands, Wang et al. further prepared a series of 2-methylallyl-based nickel complexes **30a**–**f** containing different electronic and steric substituents (Figure 27). These 2-methylallyl nickel catalysts could efficiently promote the E-MA copolymerization with high activity (**30a**: up to 17.5 × 10^3^ g (mol Ni)^−1^ h^−1^, affording copolymers with comonomer incorporation of up to 7.0 mol% [147]. Tan et al. recently explored the applications of such versatile ligands by the installation of amino/alkoxy groups to provide additional flexibilities in tuning electronic natures (Figure 28). The nickel complexes **31a**–**e** exhibited high activities (up to 1.8 × 10^5^ g mol^−1^ h^−1^) in ethylene copolymerization with a variety of polar monomers, including MA, HCl, UCOOMe, methyl 5-norbornene-2-carboxylate (NB_COOMe_) and VTMOS in the absence of any cocatalyst or protecting reagents, affording copolymers with good comonomer incorporation (1.0–4.2%) and moderate molecular weights (*M*_n_ up to 8.7 × 10^3^ Da) [148]. This electronic asymmetry strategy has also been applied to the nonalternating copolymerization of ethylene with CO, which exhibited excellent reactivity and an unprecedented nonalternating degree toward this carbonylative polymerization with a broad tolerance of organic solvents [45]. Moreover, the cationic PNPO Ni catalysts with subtle electronic variation also exhibited the remarkable productivity of 31,150 g polymer (g Ni)^−1^ for alternating copolymerization of ethylene with CO [46]. 

**Figure 29:**
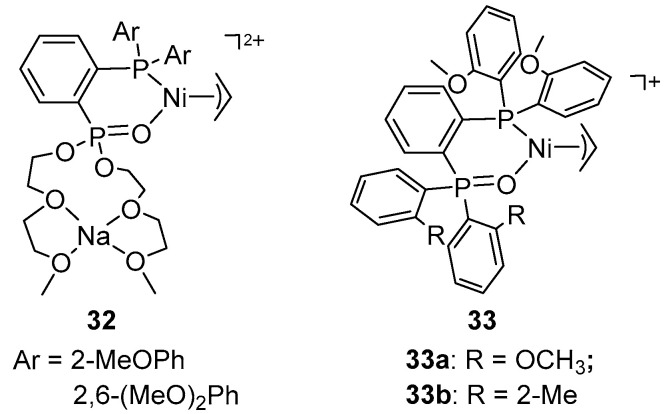
Cation-tunable **Ni** (P, O-donor) catalysts **32** and **33a**,**b** developed in the Do laboratory.

Do et al. prepared a new series of nickel phosphine phosphonate ester complexes **32** (Figure 29) that feature two metal-chelating PEG side arms [149], exhibiting enhanced catalyst effects with secondary metals (alkali, alkaline, transition, post-transition, and lanthanide ions) in studies of ethylene and polar olefin (e.g., propyl vinyl ether (PVE), BuAE, UCOOMe and PAc) copolymerization. Notably, combining either Co^2+^ or Zn^2+^ with the nickel complexes increased the rates of polymerization in the presence of PVE by about 5.0- and 2.4-fold, respectively (activity up to 34.2 × 10^3^ g mol^−1^ h^−1^ with comonomer incorporation 0.2 mol%). 

The Do group further synthesized two nickel complexes **33a**,**b** (Figure 29) featuring conformationally rigid BPMO ligands, where one had an *o*-methoxyphenyl (**33a**) and the other had an *o*-(2-methoxyethoxy)phenyl (**33b**) substituent on the P=O moiety. The addition of Li^+^ to **33b** led to clear improvements in reaction efficiency and MA per chain without diminishing the polymer molecular weight, in which a 5.4-fold enhancement in catalyst activity (9.8 kg mol^−1^ h^−1^) and a 1.9-fold increase in polar monomer incorporation (4.5 mol%) in comparison to those by **33b** alone under optimized conditions [150]. 

In comparison with classic phosphine-sulfonate ligands, the electronic and steric properties of the weak σ-donor moiety could also be modified in this BPMO ligand structure, providing a great deal of flexibilities and opportunities to tune the catalytic properties. Notably, the ligand PNPO was able to afford nickel catalysts that could copolymerize fundamental polar monomers with ethylene, which could be contributed to the smaller metal chelate-ring size and ligand rigidity. Given the various ways mentioned above to optimize such nickel complexes, we are optimistic that future breakthroughs in designing this and related systems are possible. 

## 7. *N*-heterocyclic Carbenes (NHCs) Nickel Catalysts

**Figure 30:**
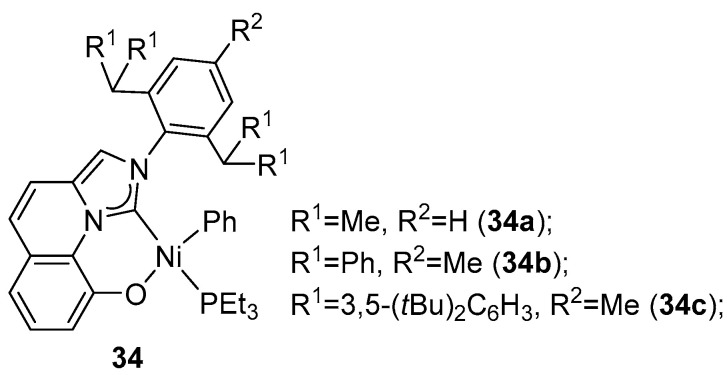
NHCs nickel catalysts **34a**–**c** bearing imidazo [1,5-a] quinolin-9-olate-1-ylidene.

The combination of a strong and weak σ-donor motif in a bidentate ligand has proved effective to suppress *β*-hydride elimination as presenting in nickel/salicylaldiminate and palladium/phosphine-sulfonate. *N*-heterocyclic-carbenes (NHCs), as the strong donor motif, are expected to have a strong σ-donating ability to enhance the electronic asymmetry in the bidentate ligands. NHC-plane and metal plane should always be coplanar, so that overlap between the vacant p-orbitals of carbene and the cis-metal-hydride is minimized, and thus catalyst decomposition via reductive elimination is retarded [151]. This idea was reflected in the rigid skeleton of palladium catalysts bearing imidazo [1,5a] quinolin-9-olate-1-ylidene (IzQO) ligands, first designed by Nozaki et al., which could act as ubiquitous catalysts for olefin polymerization to afford functionalized linear copolymers, including copolymerization of ethylene/propylene with polar-monomer [152,153,154], such as the challenging 1,1-disubstituted ethylenes [155,156]. The scope of the work was extended and nickel complexes **34a**–**c** (Figure 30) derived from IzQO ligands were also prepared which could catalyze ethylene polymerization at 50–100 °C with reasonable activity in the absence of any cocatalyst, as well as catalyze the copolymerization of ethylene with AAc and allyl ethers (AE) to obtain the corresponding copolymers with the highest molecular weight (E/AAc: greater than 4.5 kg mol^−1^) reported for a Ni-catalyzed system [27]. 

The Nozaki group recently presented an optimized synthesis of a novel type of bidentate borane/phosphine ligand and its Ni^0^ σ-borane complex, which enforced a rigid chelate through its conjugated [1,2,3] diazaborolo-[1,5a] quinoline scaffold [157]. A Ni^II^ boryl complex was further achieved by oxidative dehydrochloroborylation. This new B/P ligand allowed the nickel-catalyzed polymerization of ethylene, which could stimulate the search for novel ligand types in nickel- and palladium-catalyzed olefin polymerization reactions.

**Figure 31:**
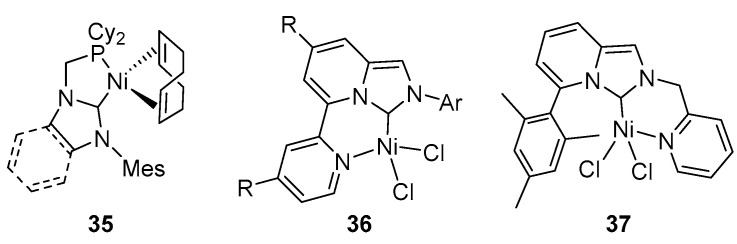
NHCs nickel catalysts **35**–**37** used in acrylate formation from ethylene and CO_2_.

Various types of nickel catalysts bearing NHCs have also been investigated in ethylene oligomerization [158,159] and norbornene (co)polymerization [160,161]. Novel nickel catalyst precursors for acrylate synthesis C–H carboxylation reaction from ethylene and carbon dioxide (CO_2_) were further explored as a highly promising sustainable process in terms of carbon capture and utilization. Iwasawa et al. prepared new zero-valent nickel complexes **35** (Figure 31) bearing an *N*-phosphinomethyl-substituted NHC ligand [162], which underwent exceptionally rapid oxidative cyclization of ethylene and CO_2_ to afford acrylate salt. The high reactivity would be associated with the higher electron density on a nickel center induced by the NHC-P ligand. Hong et al. recently presented novel mononuclear Ni(II) complexes **36** and **37** (Figure 31) with a rigid six-membered ring, imposed by a special pyridine-chelated bidentate imidazo [1,5-a] pyridine-3-ylidene ligand, providing noticeable catalytic activity in the acrylate formation from ethylene and CO_2_ (up to 108 % acrylate). The use of additional additives including monodentate phosphines increased the catalytic activity by up to 845% acrylate (TON 8) [163].

Compared with the Drent- and BPMO-type catalysts, the overall catalytic activity of *N*-heterocyclic carbene catalysts and the molecular weight of obtained copolymers were relatively low, and these catalysts were largely used in(co)polymerization of norbornene and oxidative cyclization of ethylene with CO_2_. Despite progress in recent years, there remains a long road ahead for an industrial application; therefore, more diverse ligands should be explored to develop highly efficient catalytic systems. 

## 8. Other Unclassified Nickel Catalysts

In addition to the above-mentioned major types of catalysts, the research of new frameworks has also made key progress in the past five years. Some of these catalytic systems have been introduced in the previous chapters, such as *N*-heterocyclic carbenes, diphosphazane monoxide and *N*-bridged phosphine–carbonyl Ni catalysts, which received much attention from researchers. Many other new ligands have been continuously designed to have better performance in ethylene (co)polymerization.

**Figure 32:**
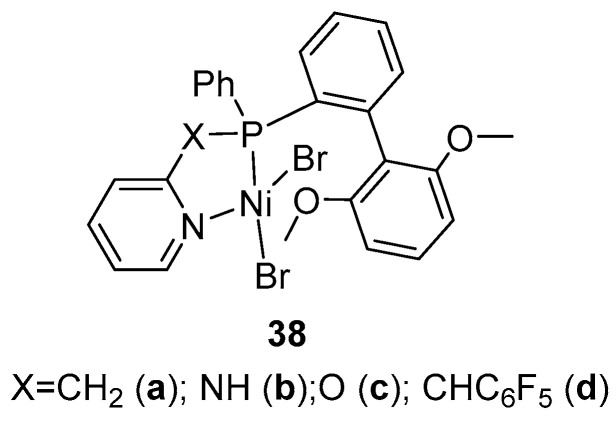
Diphenyl-substituted phosphine-pyridine catalysts **38a**–**d** with CH_2_, NH and O linkage.

Since the phosphine sulfonate and relate ligands emerged as a powerful platform in generating high-performance olefin polymerization catalysts, the Chen group reported their initial attempts by substituting the sulfonate group with a pyridine group and synthesized a series of diphenyl substituted phosphine-pyridine ligands with CH_2_, NH and O linkage [164]. The corresponding palladium catalysts **38a**–**d** (Figure 32) achieved ethylene oligomerization and cooligomerization with some polar comonomers (MA, BuVE, VTMOS, AAc and ACl), while the nickel complexes showed very high activities in ethylene oligomerization, leading to the formation of alkylated aromatic products.

**Figure 33:**
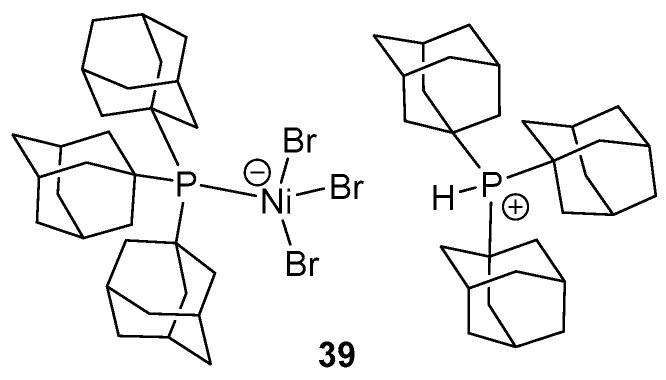
Monodentate phosphine *tri*-1-adamantylphosphine-nickel catalyst **39**.

Brookhart et al. reported a novel bulky monodentate phosphine tri-1-adamantylphosphine-nickel precursor [Ad_3_PNiBr_3_]^−^[Ad_3_PH]^+^ **39** (Figure 33) [165], which could catalyze the polymerization of ethylene to ultrahigh-molecular-weight, nearly linear polyethylene (*M*_n_ up to 1.68 × 10^6^ g mol^−1^) with initial activities reaching 3.7 million turnovers per h^−1^ at 10 °C, and copolymerize ethylene with α-olefins such as 1-hexene, 1-octadecene, and *tert*-butyldimethyl(dec-9-en-1-yloxy) silane with no decrease in activity. However, the catalyst was rapidly deactivated at higher temperatures, and both the catalytic activity and polymer molecular weight dropped sharply.

**Figure 34:**
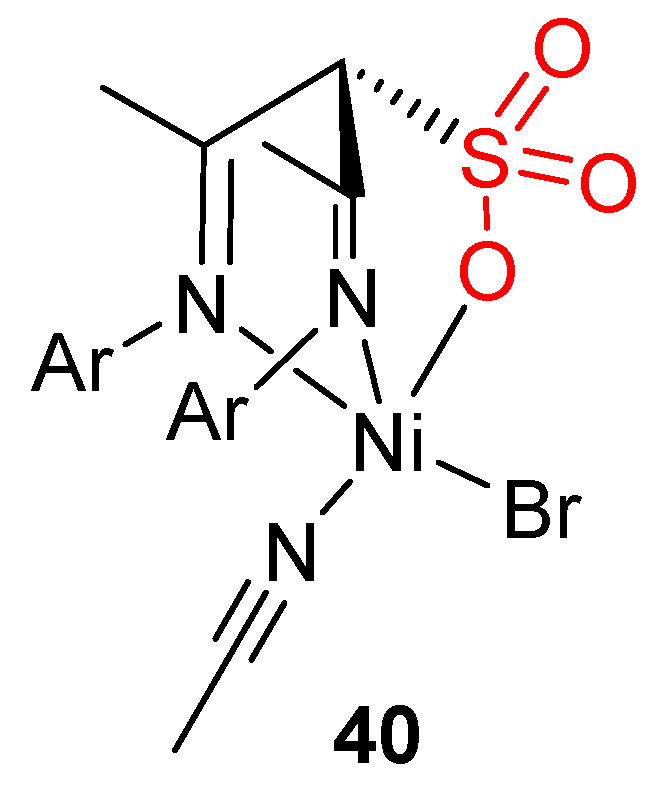
Neutral tridentate α-sulfonato-*β*-diimine nickel catalyst **40**.

By combining the advantages of cationic diimine and neutral phosphine-sulfonato type catalysts, Gao et al. designed a neutral tridentate *α*-sulfonato-*β*-diimine nickel catalyst **40** (Figure 34) with an apical SO_3_−Ni coordination in three dimensions [166]. The tridentate [N, N, O] catalyst could effectively copolymerize ethylene and MA to produce branched EMA copolymers with both high molecular weight (*M*_n_ > 20 × 10^3^) and high MA incorporation (up to 10 mol%), whose branching structure was similar to commercial EMA industrially prepared by radical polymerization under harsh polymerization conditions. DFT calculations were also performed to illuminate the formation of the EMA copolymer with terminal MA units. 

**Figure 35:**
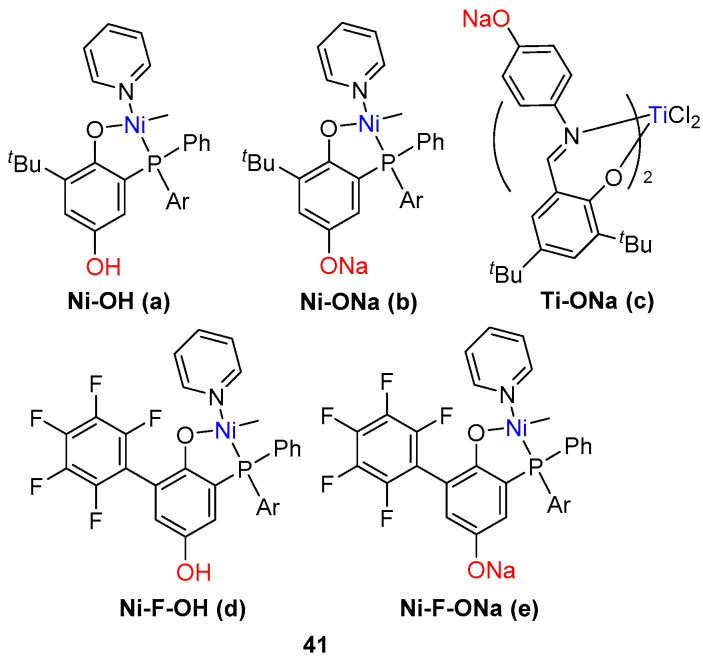
Hydroxyl-functionalized metal complexes **41a**–**e** used in the heterogeneous system.

The heterogenization of homogeneous metal complexes presents an efficient strategy for bridging homogeneous catalysts with industrially preferred heterogeneous catalysts. Chen et al. reported an alternative self-supporting strategy using simple α-diimine palladium catalysts which was amenable to gas-phase and slurry-phase polymerization techniques. Various branched ultra-high-molecular-weight PEs could be prepared, demonstrating that the utilization of chain walking could enable new polymerization methods [167]. They further developed a simple and general ionic anchoring strategy for the heterogenization of various transition metals, which enabled strong catalyst-support interactions while tolerating various polar functional groups such as MA, *t*BA and UCOOMe (Figure 35, **41a**–**e**) [168]. The heterogeneous catalysts showed greatly enhanced properties (molecular weight, incorporation, and activity) compared to their unsupported counterparts and enabled efficient polymerization at high temperatures on a large scale and great control over the product morphology.

## 9. Conclusions and Outlook

As described above, great progress in the area of nickel catalysts for olefin copolymerization has been achieved in the past three years and the performance of nickel catalysts derived from α-diimine, salicylaldimine/ketoiminato, phosphine-phenolate, phosphine sulfonates, bisphosphine monoxide, *N*-heterocyclic carbene, and their derivatives in the coordination copolymerization of functional olefins with ethylene is evaluated. More intuitively representative data for the copolymerization of ethylene and polar monomers with various types of catalysts were shown in Table 1. Though the nickel catalysts suffer from some limitations, such as generally less tolerance toward functional groups than these palladium catalysts and relatively narrow range of suitable polar monomers, the copolymerization of ethylene with the commercial polar monomers have been realized by novel ligands including cationic α-imino-ketone, phosphine-phenolate, diphosphazane monoxide, phosphine–carbonyl and heterogeneous catalysts. The microstructures of copolymers, including either linear or branched, can be well controlled by the appropriate choice of various ligands. As a promising candidate, the low-cost, earth-abundant, single-component nickel catalysts are highly desirable for industrial application, especially for the immobilization of homogeneous catalysts [64,167,168], which is crucial to preventing reactor fouling and facilitating continuous operations in industrial gas-phase and slurry processes. 

Despite its great potential, current progress still needs to overcome some critical issues such as low incorporation efficiency and insufficient studies on the structure–property relationship. It is difficult to effectively regulate the insertion rate while obtaining high-molecular-weight copolymers and precise control over the structure. Further efforts concerning nickel catalysts are still needed to address the following challenges: 

(1) Designing novel functional group tolerant catalysts through uncomplicated production processes. Various versatile strategies have been applied for ligand design, such as ligand–metal secondary interactions [37], ligand–monomer interactions [39], ancillary ligand (polar additives) regulation [119,169,170], and multi-nuclear synergistic effects [50,99]. The backbone/framework structure and the substitution groups on the ligands are of great importance in determining the catalytic properties, and further catalyst development should be targeted toward a better balance between the polymer molecular weight, incorporation, and catalytic activity. It is indispensable for addressing related issues by designing easily available, relatively cheap, and high-efficiency catalysts.

(2) Broaden the scope of the variety of polar monomers suitable for the copolymerization. Except for the challenging fundamental comonomers, disubstituted polar vinyl monomers and those abundant and less expensive ones as well as the incorporation of renewable functional olefins [134,171] with ethylene also opens up new avenues and offers distinct advantages, which could give novel copolymers with unique physical and chemical properties, such as antibacterial, antioxidant, dynamic cross-linking, self-healing, shape memory, photodegradability, and recoverable catalyst support.

(3) Designing new polymerization modulation strategies. An alternative strategy that has emerged in recent years is the application of tunable catalysts in polymerization that could be toggled between different reactivity states in response to external stimuli, such as redox [36], electrochemical [172], photochemical [73,173,174], secondary metal-binding [38,90,175] and supramolecular chemistry [176] control. Furthermore, the heterogeneous strategy is highly promising for the further identification of applications in different catalytic systems and the synthesis of advanced engineering materials.

(4) Characterization of the physical and chemical properties of the obtained polymer materials. For industrial applications, the studies on material properties (viscosity, rheology, surface, mechanical, etc.) and their relationship with catalyst structures are also critical. Moreover, the lack of mechanism studies on nickel catalysts compared with the corresponding palladium limit their further applications. Several competing reactions may occur in copolymerization simultaneously, and each catalyst may have multiple deactivation and decomposition pathways. It is necessary to explore the reaction mechanism and clarify the pathway, type, and formation process of copolymer microstructures, and to elucidate the real influencing parameters to realize the modulation of copolymer properties.

The development of late transition nickel catalysts is timeless in the olefin (co)polymerization reaction, and many challenges, opportunities, and possibilities still remain in this field. Thus, substantial potential exists for innovation in functional polyolefin synthesis from the aspects of novel catalyst and polar monomer design. We will see a bright future for nickel-mediated copolymerization in industrial production.

## Figures and Tables

**Table 1:** Representative data for the copolymerization of ethylene and polar monomers with various types of catalysts.

Catalyst	Polar Monomers Used	Activity (g mol^−1^ h^−1^)	*M*_w_ Range (g mol^−1^)	Polymer Characteristics	Refs.
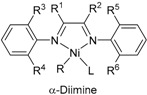	UCOOMe, UCOOH, UOH, UCl, HO, VTMOS, HCl	~10^5^	10^4^~10^5^	Branches	[66,67,68,69,70,71,72,73]
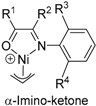	HCl, UCOOMe, MA	10^4^~10^6^	10^3^~10^5^	Branches	[22,95,96]
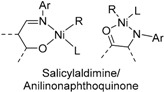	HAc, AAc, AAA, VA, AA	10^4^~10^6^	10^3^ ~ 10^5^	Branches	[79,93,94,99]
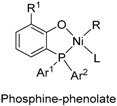	MA, BA, *t*BA, DMAA, MMA, NVP, VTMOS, HCl, BuAE,	10^3^~10^5^	~10^4^	Low branches	[108,109,111]
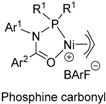	UCOOMe, AA MA, AAc, ACl, BuVE	10^3^~10^5^	10^3^~10^4^	Low branches	[113,114,115,116]
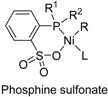	HCl, UCOOMe, UCOOH, UOH VTMOS, Norbornenes	10^4^~10^5^	10^3^~10^4^	Low branches to linear	[120,121,122,123,124,127,128,129]
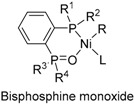	UCOOMe, HCl, PAc, PVE, BuAE	10^3^~10^5^	10^3^~10^4^	Linear	[144]
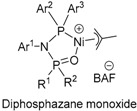	MA, HCl, UCOOMe, NB_COOMe_, VTMOS	10^3^~10^5^	~10^3^	Linear	[145,147,148]
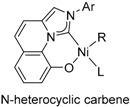	AAc, AE	10^2^~10^3^	~10^3^	Linear	[27]
